# Reflections on *SICOT-J* Volume 11 (2025)

**DOI:** 10.1051/sicotj/2026006

**Published:** 2026-04-29

**Authors:** Raju Vaishya, Sébastien Lustig, Andreas F Mavrogenis, Vikas Khanduja

**Affiliations:** 1 Department of Orthopaedics and Joint Replacement Surgery, Indraprastha Apollo Hospitals Sarita Vihar New Delhi 110076 India; 2 Orthopaedics Surgery and Sports Medicine Department, FIFA Medical Center of Excellence, Croix-Rousse Hospital, Hospices Civils de Lyon, Lyon North University Hospital, 69004 Lyon, France - Univ Lyon, Claude Bernard Lyon 1 University, IFSTTAR, LBMC UMR_T9406 69622 Lyon France; 3 First Department of Orthopaedics, National and Kapodistrian University of Athens, School of Medicine 15562 Athens Greece; 4 Department of Trauma & Orthopaedics, Addenbrooke’s – Cambridge University Hospital Cambridge UK

## Abstract

Volume 11 (2025) of *SICOT-J* showcases high-quality global orthopaedic research spanning spine, trauma, arthroplasty, sports, and perioperative care. Through impactful original studies, reviews, and editorials, the volume reinforces evidence-based practice, surgical innovation, and multidisciplinary approaches to contemporary musculoskeletal challenges worldwide.

Volume 11 (2025) of *SICOT-J* illustrates the journal’s pivotal role in disseminating open-access orthopaedic research. This volume’s diverse portfolio, encompassing original research, systematic reviews, technical notes, and editorials, reflects both the global membership of the *Société Internationale de Chirurgie Orthopédique et de Traumatologie* (SICOT) and the evolving needs of orthopaedic surgeons and researchers worldwide.

A hallmark of this volume was its focus on arthroplasty outcomes. Multiple original studies investigated total knee and hip arthroplasty procedures, not only evaluating standard outcomes but also interrogating nuanced clinical factors. For example, research on oxidative stress modulation in total knee arthroplasty (TKA) suggests promising avenues for biochemical optimization of perioperative physiology, a novel adjunct to conventional care. Similarly, long-term outcome data from patients who experienced intraoperative periprosthetic femoral fractures during cementless hip hemiarthroplasty challenge traditional assumptions about the prognostic impact of such adverse events, opening debate on tailored intraoperative management strategies.

In the spine section, an original investigation, “*Validity of a new scoring system for assessment and decision guidance of misplaced pedicular screws*”, introduces a novel Meshtawy Pedicular Screw Malposition (MPSM) score, demonstrating excellent intra- and inter-observer reliability and promising clinical decision support for screw misplacement management [1]. Complementing this, the comprehensive review “*Osteoporotic vertebral fractures: an update*” by Daskalakis et al. [2] synthesizes current evidence on diagnosis, management, and secondary prevention, emphasizing the urgent need for vigilant detection and multidisciplinary care in fragility fracture populations.

Important contributions were made in the field of knee research. Mustamsir et al.’s updated systematic review, “*Clinical outcomes and long-term efficacy of high tibial osteotomy in treating knee instability*”, aggregates existing evidence on high tibial osteotomy, highlighting its role in restoring stability and potentially delaying more invasive interventions [3]. In addition, articles such as “*Venous thromboembolism prophylaxis after anterior cruciate ligament reconstruction: retrospective case-control study*” by Afana et al. [4] examine perioperative complications and risk mitigation; a topic of ongoing importance in sports and trauma surgery.

The shoulder section features “*Therapeutic options in rotator cuff calcific tendinopathy*” by Moya and co-authors [5], a clinically valuable review that evaluates treatment alternatives such as Extracorporeal Shock Wave Therapy (ESWT) and ultrasound-guided barbotage for a condition that remains a therapeutic challenge.

This volume also highlights innovations in joint arthroplasty and perioperative care. Among several original reports, “*Direct anterior total hip arthroplasty with dual mobility cup for femoral neck fractures in dementia patients*” by Okuno et al. [6] explores functional outcomes in a vulnerable, often underreported subgroup of hip fracture patients. Other studies assess perioperative blood management and graft harvest techniques, such as “*Superficial band of the quadriceps tendon harvested with a minimally invasive technique provides adequate graft dimensions: a cadaveric study*” [7], further enriching the evidence base for surgical technique optimization.

This volume also includes a thought-provoking editorial, “*Analgesia considerations in orthopaedic surgery: the role of magnesium sulfate infusions*” by Papadimos et al. [8], which discusses magnesium sulfate as an adjunct to multimodal pain management; a topic that resonates with perioperative care goals across subspecialties. Bagaria and Vaishya, in *“Transforming joint replacement with open robotics: A call for change!”* [9], compellingly advocate for open robotic platforms in arthroplasty, highlighting their potential to democratize technology, enhance surgical precision, and foster innovation through transparency and collaborative development.

Looking ahead to 2026, *SICOT-J* has announced special thematic foci that will further broaden its scholarly impact. A forthcoming “*Paediatric Orthopaedics*” issue will collate cutting-edge clinical and translational work dedicated to musculoskeletal conditions in children, reflecting the growing global emphasis on tailored care from infancy through adolescence. Additionally, a “*Technology in Orthopaedics*” special issue is anticipated to explore innovations such as robotics, artificial intelligence (AI), navigation, and digital health tools that are reshaping surgical practice and patient outcomes. These focused collections will provide timely, high-impact insights at the intersection of subspecialty care and emerging technology, reinforcing *SICOT-J*’s role as a leader in orthopaedic scholarship.

We believe that the continued integration of multidisciplinary perspectives, robust clinical cohorts, and translational insights will be essential for further advancing patient outcomes and strengthening *SICOT-J*’s impact in the orthopaedic community.

## Data Availability

This article has no associated data generated and/or analyzed.
